# *Streptococcus suis*: An Underestimated Emerging Pathogen in Hungary?

**DOI:** 10.3390/microorganisms8091292

**Published:** 2020-08-24

**Authors:** Márió Gajdács, Anita Németh, Márta Knausz, Ibrahim Barrak, Anette Stájer, Gyula Mestyán, Szilvia Melegh, Adrienn Nyul, Ákos Tóth, Zsuzsanna Ágoston, Edit Urbán

**Affiliations:** 1 Department of Pharmacodynamics and Biopharmacy, Faculty of Pharmacy, University of Szeged, Eötvös utca 6., 6720 Szeged, Hungary; 2 Institute of Medical Microbiology, Faculty of Medicine, Semmelweis University, Nagyvárad tér 4., 1089 Budapest, Hungary; 3 Microbiology Laboratory, Petz Aladár County Teaching Hospital, Vasvári Pál utca 2–4., 9023 Győr, Hungary; anita8909@hotmail.com (A.N.); knauszm@petz.gyor.hu (M.K.); 4 Department of Prosthodontics, Faculty of Dentistry, University of Szeged, Tiszta Lajos körút 62–64., 6720 Szeged, Hungary; barrakibrahim@gmail.com; 5 Department of Periodontology, Faculty of Dentistry, University of Szeged, Tiszta Lajos körút 62–64, 6720 Szeged, Hungary; stajeranette@gmail.com; 6 Department of Medical Microbiology and Immunology, Faculty of Medicine, University of Pécs, Szigeti út 12., 7624 Pécs, Hungary; mestyan.gyula@pte.hu (G.M.); melegh.szilvia@yahoo.co.uk (S.M.); nyul.adrienn@pte.hu (A.N.); 7 Department of Bacteriology, Mycology and Parasitology, National Public Health Center, Albert Flórián út 2–6., 1097 Budapest, Hungary; toth.akos@nnk.gov.hu; 8 Department of Anaesthesiology and Intensive Therapy, Faculty of Medicine, University of Szeged, Semmelweis utca 6., 6725 Szeged, Hungary; agoston.zsuzsanna@med.u-szeged.hu; 9 Institute of Translational Medicine, Faculty of Medicine, University of Pécs, Szigeti út 12., 7624 Pécs, Hungary; zsoldiedit25@gmail.com

**Keywords:** *Streptococcus suis*, zoonosis, emerging infection, surveillance, meningitis, sepsis, hearing loss, agriculture, Hungary

## Abstract

*Streptococcus suis* (*S. suis*) is an emerging zoonotic pathogen, demonstrated as an etiological agent in human infections in increasing frequency, including diseases like purulent meningitis, sepsis, uveitis-endophtalmitis and arthritis. Due to the increased availability and utility of novel diagnostic technologies in clinical microbiology, more studies have been published on the epidemiology of *S. suis*, both in veterinary and human medicine; however, there are no comprehensive data available regarding human *S. suis* infections from East-Central European countries. As a part of our study, data were collected from the National Bacteriological Surveillance (NBS) system on patients who had at least one positive microbiological result for *S. suis,* corresponding to an 18-year study period (2002–2019). *n* = 74 *S. suis* strains were isolated from invasive human infections, corresponding to 34 patients. The number of affected patients was 1.89 ± 1.53/year (range: 0–5). Most isolates originated from blood culture (63.5%) and cerebrospinal fluid (18.9%) samples. Additionally, we present detailed documentation of three instructive cases from three regions of the country and with three distinctly different outcomes. Hungary has traditional agriculture, the significant portion of which includes the production and consumption of pork meat, with characteristic preparation and consumption customs and unfavorable epidemiological characteristics (alcohol consumption, prevalence of malignant diseases or diabetes), which have all been described as important predisposing factors for the development of serious infections. Clinicians and microbiologist need to be vigilant even in nonendemic areas, especially if the patients have a history of occupational hazards or having close contact with infected pigs.

## 1. Introduction

*Streptococcus suis* (*S. suis*) is an emerging zoonotic pathogen, which has been demonstrated as an etiological agent in human infections in increasing frequency [1,2]. The clinical presentations of these—often serious or life-threatening—infections may be wide-ranging, including syndromes such as purulent meningitis, sepsis, streptococcal toxic shock-like syndrome (STSLS), uveitis-endophthalmitis and arthritis; nevertheless, *S. suis* has also been described as an opportunistic pathogen of lower respiratory tract infections (e.g., pneumonia) [3,4,5]. In recent years, due to the increased availability and utility of novel diagnostic technologies and molecular biological methods both in veterinary and human clinical microbiology, more studies have been published on this zoonotic infectious entity, including the epidemiology of human infections and the virulence factors of this pathogen [6,7]. The bacterium has been described as a colonizing agent in the upper respiratory, gastrointestinal tract, and genital organs of various animals (pigs, ruminants, cats, dogs, horses and other wild animals). *S. suis* was first reported in 1954 by veterinarians after outbreaks of septicemia and/or meningitis and purulent arthritis occurred among piglets [8,9,10]. Fourteen years later, some human *S. suis* cases were first described in Denmark [11]. So far, over 1600 human cases have been reported in the literature worldwide, although this infection is probably severely underdiagnosed [1,2,12]. Subsequently, the number of human *S. suis* cases reported has increased over the past few years, with the highest prevalence rate in Southeast Asia, where there is a high rate of pig meat consumption: the majority of these infections originated from Thailand (~36%), Vietnam (~30%) and China (~22%); therefore, Thailand and Vietnam have the highest disease prevalence stratum globally, with estimated 0.82 and 0.54 cases/100,000 population, respectively [13,14]. In contrast, the prevalence in Western European countries ranges between 0.01–0.10 cases/100,000 population (with highest prevalence in the Netherlands and the UK) [1,4]. The epidemiological situation in Australasia is similar to the one on the European continent, while the Americas and Japan has reported the lowest prevalence of human *S. suis* cases (<1% of global cases) [1,2,3,4]. The clinical relevance of these invasive infections must not be underestimated; based on the results of a meta-analysis, the mortality rate associated with these infections is 12.8% (confidence interval: 9.0%–18.0%), with notable geographical differences observed among geographical regions (Asia: up to 26%, Western countries: 2–4%); in addition, almost half of the patients experience some kind of neurological sequelae after recovery [4].

These bacteria are facultatively anaerobic Gram-positive, ovoid or coccal bacilli, with sizes on average between 1.0 to 1.5 µm, appearing in pairs, short chains or as single bacteria [15]. The bacterial species is genetically and phenotypically very heterogeneous, which were earlier classified into the *Streptococcus* Lancefield groups R, S, and T [16]. Currently, *S. suis* is classified within the family *Streptococcaceae*, order *Lactobacillales*, phylum *Firmicutes* [17]. The bacterial virulence-associated factors may be classified into the following sub-groups (of note, some genes probably may be classified into more than one categories due to their dual characteristics): (i) surface/secreted elements; (ii) protease enzymes; (iii) transcription factors/regulatory systems and (iv) other virulence determinants (transporters/secretion systems) [18]. The list of confirmed and putative virulence factors is extensive, but the most prominent and best-characterized virulence factors of clinical *S. suis* strains are surface and secreted elements: capsular polysaccharides, extracellular factor (EF; *epf*), muramidase-released protein (MRP; *mrp*) and a 38-kDa surface protein, suilysin (SLY) [19,20,21,22]. *S. suis* isolates are included a complex population consisting of heterogeneous strains and they may be classified into 35 serotypes (1–34, 1/2) to date, based on the principal virulence factor, the capsular polysaccharide antigen (CPS) [23,24]. Human *S. suis* cases are reported to be mainly due to serotype 2 (SS2) (24.3–74.7%) and 14 (2.0–13.0%), with both serotypes being equally implicated in cases of meningitis (50–70%) and sepsis (20–25%) [4,25]. SS2 is believed to be the most virulent type, with a polymorphic mosaic genome, which is associated with a higher virulence and is frequently isolated from humans affected by these infections [26]. The occurrence of serotype 14 infections was sporadically reported in Northern Thailand, Vietnam and case reports were also described in the UK, France, Australia, and Canada [27,28]. In constrast, serotype 9 is one of the most common (19.4–61.0%) in the pigs of some Western European countries [29]. Infections caused by serotypes 4,5,8,9,11,12,13,16,21,24,31 and species with untypeable serotypes are considered to be rare (0–2.7%)—only sporadic cases were reported [4,30,31,32,33,34,35]. *S. suis* isolates causing streptococcal toxic shock syndrome (STSS) and high mortality rates were associated with the presence of a pathogenicity island (89K PAI), most commonly seen in large Chinese outbreaks [36].

There are some typical risk factors for acquiring *S*. *suis* infections such as raw (or uncooked) pork consumption, direct exposure to pigs or pork, workplace exposure in several professions (e.g., butchers, slaughterhouse workers, hunters, retail workers in butcheries), excessive consumption of alcohol, skin injuries (especially during handling pig or pork), male gender and some underlying diseases contributing to immuno-compromised conditions [37,38]. Most patients, who were infected by serotypes other than SS2, had liver cirrhosis [30,34] or other pre-existing immunosuppressive states [39]. In addition, as previously reported for invasive Group B *Streptococcus* infections, alcoholism, diabetes mellitus, splenectomy and malignancies have been suggested as important predisposing factors for the development of rapid and fatal *S. suis* infections [40]. In humans, the intestinal route of infection seems to be an important port of entry, after consumption of fresh/raw contaminated pork meat (e.g., in some Southeast Asian countries) [41]. In fact, the epidemiology of human infections may be significantly affected by the cultural differences and eating habits between the Asian and the European/American regions, while this also raises concerns regarding imported cases due to increasing levels of tourism towards Asia [1,2,3,4,5]. Apart from the handling or ingestion of pork meat, direct contact with the mucous membranes of pets (cats, dogs), livestock (horses, ruminants) and wild animals (deer, wild boar) may also be an important source of infection [37,38]. To date, human-to-human transmission has not been demonstrated to occur. Nevertheless, the transmission of bacteria through skin abrasions was also believed to be an important route of infection, altough skin injuries in patients during exposure or before infection were noted only in some studies [42]. Earlier studies suggest that sometimes humans could be healthy carriers of *S. suis* as well [43,44,45]. Encapsulated *S. suis* strains are highly invasive pathogens; after penetration of host mucosal barriers, these bacteria may reach and survive in the blood cells and they may subsequently invade different organs, such as liver, kidney, spleen, lung and even the heart [46]. Pathogenic strains are able to cross the brain microvascular endothelial cells (BMECs) and/or the epithelial cells of the choroid plexus at the blood–brain barrier (BBB) and/or the blood–cerebrospinal fluid barrier to gain access to the central nervous system (CNS) [43,47,48,49]. After an incubation period that ranges from a few hours to a few days in humans, *S. suis* infections result in meningitis, sepsis, pneumonia, endocarditis or peritonitis [1,2,12,43,50,51]. In addition, peracute infections with shock and a high mortality rate have been described, particularly in the case of streptococcal toxic shock-like syndrome (STSLS) [52].

Around 8–10% of cases worldwide were reported from the European continent; however, to date, there are no comprehensive epidemiological data available in the literature regarding human *S. suis* infections from East-Central European countries [4,15,53]. The aim of our present study was to report on the emergence and clinical manifestations of *S. suis* infections in Hungary over a long surveillance period—using data from the national bacteriological database—in addition to illustrating the various possible clinical outcomes of human *S. suis* infections through the description of instructive cases from various Hungarian clinical centers.

## 2. Materials and Methods

### 2.1. Study Site and Population

Hungary is a medium-sized, landlocked country in East-Central Europe, with a total area of 93,030 km^2^ and a population of 9,772,756 persons (estimated from the most recent census data; population density: 105.1/km^2^) [54]. The area of Hungary may be divided into 7 regions or 19 counties (in addition to the capital of Budapest) for administrative purposes. Based on Organisation for Economic Co-operation and Development (OECD) guidelines, Hungary is a high-income country (with the 54th largest global economy based on nominal gross domestic product (GDP) and 45th highest Human Development Index (HDI)) [55]. The average life expectancy in Hungary is 72.4 years for males and 79.0 years for females. Hungary currently has 165 hospitals, with 70 beds and 40.9 medical doctors per 10,000 people [56].

Among national characteristics pertaining to risk factors of *S. suis* infections, 28.65% of the population lives in rural areas and around 5% of the population works in full-time agriculture [56,57]. According to the census data, 52.4% of the population is female [54]. Hungary has one of the highest rates of people with type II diabetes, very high rates of smoking prevalence and one of the highest rates of morbidity due to malignant diseases in Europe [54,55,56,57]. The total per capita consumption of alcohol is estimated to be 11.4 L/year, which is much higher than the worldwide average [~6 L/year], based on WHO criteria [58]. Meat consumption is estimated to be 65.04 kg/person annually (moderate), out of which ~25 kg/person (i.e., 38.5%) is pork meat per capita [59]; however, this ratio was over 50% during the period between 2000 and 2010. In an attempt to boost domestic pork production and consumption, the Hungarian government has cut the value-added tax (VAT; from 27% to 5%) on pork offal and related products [60].

### 2.2. Data Collection

As a part of our study, data were collected from the Subcommittee on Gram-positive bacteria of the National Bacteriological Surveillance (NBS) system on patients who had at least one positive microbiological result for *S. suis* in a hospital (including intensive care units and departments of traumatology, surgery, pediatrics, dermatology, ophthalmology, obstetrics and gynecology, otorhinolaryngology, and head and neck surgery) in Hungary, between 1 January 2002 and 31 December 2019 (18-year-long period) [61]. The following anonymized data were exclusively collected during the study: age at sample submission, gender, place of residence, type of sample(s) submitted for microbiological analysis, results of microbiological analyses, and antimicrobial susceptibility testing.

### 2.3. Bacterial Identification, Antibiotic Susceptibility-Testing

Sample processing and microbial identification in the respective microbiology laboratories were carried out according to guidelines for routine clinical bacteriology. Cerebrospinal fluid samples and blood samples were taken for both clinical chemistry and microbiology. Blood culture samples for microbiological analysis were inoculated into blood culture bottles and incubated in automated blood culture systems. If the blood culture instrument reported a positive result, samples were inoculated onto relevant agar media. In case of cerebrospinal fluid samples, enrichment broth media were also used in addition to agar media. Latex agglutination-based rapid tests—aiming to identify the main pathogens of bacterial meningitis from the cerebrospinal fluid—were also performed. After 2013, several tertiary-care hospitals and specialized-care centers have introduced matrix-assisted laser desorption/ionization time-of-flight mass spectrometry (MALDI-TOF MS) into their diagnostic workflow [62,63].

Antimicrobial susceptibility testing was performed by the disk diffusion method for penicillin (from which, susceptibility to other β-lactams may be inferred), erythromycin, clindamycin, moxifloxacin, tetracycline, and vancomycin. Inducible clindamycin resistance was detected using the D-test and these strains were also reported as resistant [64]. The results were interpreted following the breakpoints for viridans streptococci approved by the European Committee on Antimicrobial Susceptibility Testing (EUCAST) for penicillin and other β-lactam antibiotics, clindamycin and vancomycin, Clinical and Laboratory Standards Institute (CLSI) guidelines were used for erythromycin and tetracycline, while in the case of moxifloxacin, *S. pneumoniae* breakpoints were used [4,15]. Intermediate results were grouped with and reported as resistant.

### 2.4. Statistical Analysis

Due to the low number of relevant isolates, only descriptive statistical analyses were performed. Categorical variables were summarized by frequencies and percentages, while continuous data were presented as mean ± standard deviation (SD) and counts or percentages (%). All statistical analyses were performed using Statistical Package for Social Science (SPSS) software (IBM SPSS Statistics for Windows 24.0, IBM Corp., Armonk, NY, USA).

### 2.5. Ethical Statement

Data collection on anonymized patient affected by *S. suis* infections was in accordance with ethical standards at the institutional and/or national research committees and with the 1964 Helsinki Declaration and its later amendments.

## 3. Results

### 3.1. Epidemiology and Antibiotic Susceptibility of S. suis Infections in Hungary

**Figure 1:**
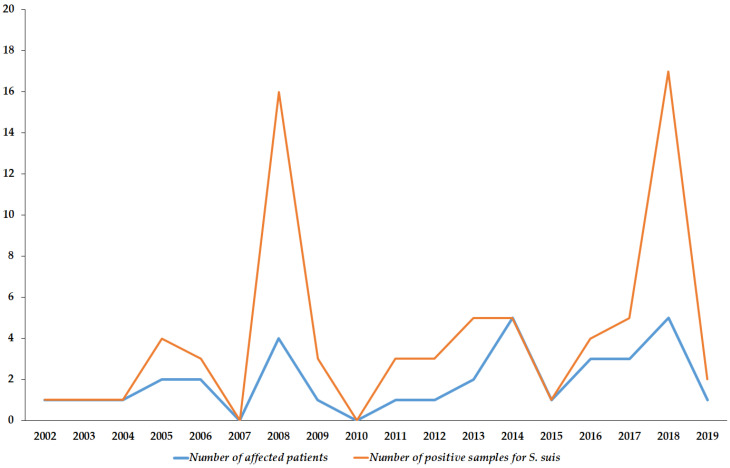
Secular trends of the isolation of *S. suis* in Hungary (2002–2019).

**Figure 2:**
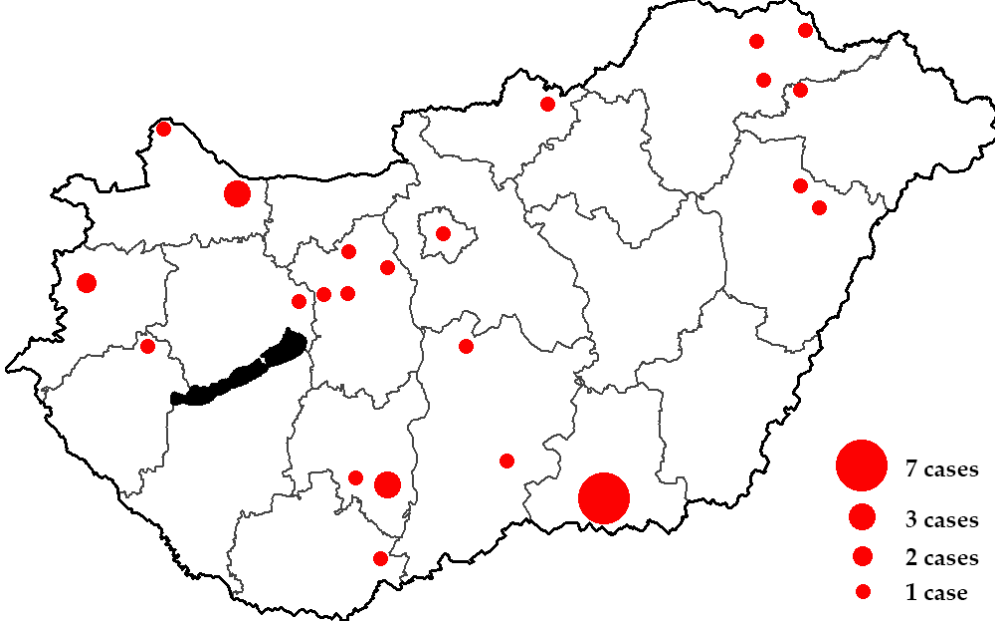
Spatial distribution of human *S. suis* clinical cases in Hungary (2002–2019).

During the 18-year study period (2002–2019) *n* = 74 *S. suis* strains were isolated from invasive human infections in Hungarian hospitals (based on reports to the NBS), corresponding to 34 patients. The number of affected patients was 1.89 ± 1.53/year (range: 0–5), which was consistent throughout the study period; however, there were zero cases in 2007 and 2010, while peaks of *n* = 5 cases were seen in 2014 and 2018, respectively. The temporal distribution of *S. suis* infections is presented in Figure 1, while the spatial distribution of cases in Hungary is shown in Figure 2. The number of cases was almost double as high in the second half (2011–2019) of the study period (12 vs. 22). The average incidence of *S. suis* infections was 0.35/100,000 persons. The median age of affected patients in 57 years (range: 21–91 years), while 20 out of 34 patients (58.8%; male-to-female ratio: 1.43) were males. Among the indications listed for sample submissions, the most common was meningitis (*n* = 36, 48.6%), followed by fever (*n* = 18, 24.4%), sepsis (*n* = 10, 13.5%), and other infectious processes (*n* = 10, 13.5%). Immuno-suppression was verified in only 5 out of 34 patients, while a known history of contact with pigs or other companion animals was noted only in 4 out of 34 patients.

Out of the *n* = 74 samples positive for *S. suis*, *n* = 47 (63.5%) were blood culture samples, *n* = 14 (18.9%) were cerebrospinal fluid samples, *n* = 6 (8.1%) were wound samples, while *n* = 7 (one abscess sample, one bile samples, one inner ear aspirate, two pus samples and two intra-abdominal fluid samples; 9.5%) were others. The incubation period usually ranged between <1 to 4.2 days. In case of *n* = 7 patients, *S. suis* was simultaneously present in both their blood culture and cerebrospinal fluid samples. During the analysis of susceptibility-testing results, only the first isolate per patient was included: all the respective isolates were uniformly susceptible to β-lactam antibiotics and vancomycin (34/34), while 14/34 of isolates were resistant to erythromycin and clindamycin, 10/34 of isolates were resistant to moxifloxacin and 9/34 were resistant to tetracycline. Data on *S. suis* serotypes were only available in the in case of the most recent 11 cases (including the three cases described in detail below): *n* = 10 were the SS2, while *n* = 1 was the SS14 serotype.

### 3.2. Description of Three Distinct Cases in Hungary with Remarkably Different Clinical Outcomes

To further illustrate the importance of *S. suis* infections in various clinical situations, herein we present detailed documentation of three instructive cases from the material of the last two years, from three regions of the country and with three distinctly different outcomes for each affected patient.

#### 3.2.1. Case 1

At the Petz Aladár County Teaching Hospital in Győr in January 2018—in the midst of an influenza epidemic—a male patient presented with symptoms of purulent meningitis, whose brief medical history is described below: the 48-year-old male patient was admitted to the Emergency Department (ED) after being transferred to the hospital by the National Ambulance Service from the patient’s place of residence. The patient was admitted due to a headache; high fever (41 °C) and myalgia, which presented two days before; and vomiting, diarrhea and aphasia, which presented one day before hospital admission. The past medical history of the patient was unremarkable. The vital signs of the patient were the following: 156/104 mmHg, pulse: 117/min, body temperature: 37.3 °C (due to administration of antipyretic medication at home), oxygen saturation: 98%. Upon physical examination, the patient was alert but disoriented, meningeal excitatory signs (Kernig, Brudzinski) were positive. On admission, other abnormalities were not detected; a rash, bleeding, swollen or painful joints were not seen. Among laboratory parameters, the WBC count of 25.6 G/L and the CRP-levels of 162.4 mg/L were of interest. No abnormalities were seen on the chest X-ray or the head CT. Subsequently, samples were taken for laboratory analysis: blood samples and cerebrospinal fluid were sent to the Department of Clinical Chemistry, while two sets of blood culture bottles and a cerebrospinal fluid sample was sent to the microbiology department for culture. During the lumbar puncture, opalescent cerebrospinal fluid was observed, flowing at medium pressure. The result of the examination of the cerebrospinal fluid at the Department of Clinical Chemistry was the following: cell count: 750, protein: 3.5 g/L, glucose: 1.2 mmol/L. Rapid tests from the cerebrospinal fluid, aiming to identify the main pathogens of bacterial meningitis (*S. pneumoniae*, *S. agalactiae*, *Haemophilus influenzae*, *Neisseria meningitidis* A, C, Y, W135, *N. meningitidis* B, *E. coli* K1) showed a negative result. A Gram-stained smear from the cerebrospinal fluid was observed under a microscope: 20–30 granulocytes and Gram-positive, spear-shaped diplococci were observed per field of view. Based on the microscopic image, the pathogenic role of *S. pneumoniae* was suspected, but this was not confirmed by the rapid latex agglutination test. Following the administration of dexamethazone, empiric antibiotic therapy (ceftriaxone, ampicillin) was initiated at the ED, according to the protocol for the treatment of community-acquired purulent meningitis, and then the patient was placed in the Infectious Disease Ward. On the second day of treatment, an α-hemolytic Streptococcus was cultured from both the blood culture samples and the cerebrospinal fluid. Identification was carried out using an ID32 Strep (bioMérieux, Marcy-l’Étoile, France) semi-automatic identification system; the pathogen has been successfully identified as *S. suis*. For verification purposes, the isolate was sent to the National Institute of Public Health; the original identification was also verified by a MALDI-TOF MS system.

Antibiotic susceptibility testing of the isolate was carried out: the isolate was shown to be susceptible to penicillin and ceftriaxone. Armed with susceptibility-results, the empiric ampicillin-therapy was suspended and ceftriaxone monotherapy was initiated which lasted for 14 days. Consultation with a cardiologist has excluded the possibility of endocarditis. Audiological examinations were not carried out, as the patient did not have such symptoms. After the administration of the antibiotic therapy, the patient’s complaints have disappeared, the laboratory parameters regressed, and the control microbiological examinations (blood cultures, cerebrospinal fluids) were negative. The isolate was found to be the SS2 serotype. No sequelae were observed either after discharging of the patient or after follow-up examinations one month later. Given the role of *S. suis* in zoonosis, a detailed heteroanamnesis revealed that the patient kept Mangalica (Mangalitsa or Mangalitza), a Hungarian breed of domestic pigs, on his family farm and one of his hobbies included hunting. Two days before he fell ill, he hunted down a wild boar and processed it in his own home.

#### 3.2.2. Case 2

A 37-year old male patient—who was employed as a waste collector—was transferred from the ED of the local hospital in Mohács to the Department of Neurology of the University of Pécs, due to progressive loss of consciousness, headache, fever, numbness of the right arm and primary progressive aphasia. The patient’s past medical history was unremarkable, apart from known diagnoses of ulcus duodeni and hypertension. Based on the reports of the ambulance staff, the socio-economic status of the patient was low (in their apartment, a high degree of clutter and garbage was seen). The patient showed meningeal excitatory signs (Kernig, Brudzinski) and presented with neck stiffness; thus, blood cultures and cerebrospinal fluid samples were taken for microbiological analysis. During laboratory analysis, inflammatory markers (C-reactive protein: 200 mg/L, WBC: 18,700/µL) were elevated and 60,000–70,000/mm^3^ of granulocytes were counted in the cerebrospinal fluid. Results of the latex-agglutination “meningitis” rapid tests were negative. Both samples types were positive for an optochin-resistant, α-hemolytic Streptococcus, which was subsequently identified as Aerococcus viridans by an API 20 Strep Kit (bioMérieux, Marcy-l’Étoile, France). As the patient did not have any underlying illnesses that would point to the infectious role of A. viridans, the bacterial isolates were sent to two partnering laboratories for further processing. In the Bay Zoltán Nonprofit Ltd. for Applied Research (Budapest, Hungary), MALDI-TOF MS analysis was carried out, which has identified the strain as *S. suis* with a reliable log score (>2.300). In the Department of Epidemiology and Microbiology, Faculty of Veterinary Medicine, Szent István University (Budapest, Hungary), two methods were utilized; namely, the Biolog MicroStation™ ID System (Hayward, CA, United States), and 16S rRNA gene sequencing (Illumina, San Diego, CA, United States). While the former method was unable to identify the isolate in question, 16S rRNA gene sequencing has found the highest (99%) match with the sequences of *S. suis*. The isolate was the SS2 serotype of *S. suis*. After successful identification, antibiotic susceptibility testing was carried out: the isolate was susceptible to β-lactam antibiotics, therefore the empirical ceftriaxone therapy was continued and the patient’s condition improved rapidly. The patient was discharged after 14 from his initial admittance to the ED. The patient denied any sort of contact with pigs or other companion animals. During follow-up examinations, the patient was assessed at the Department of Oto-Rhino-Laryngology, where the patient reported deafness on the right side, and perceptional hearing loss on the left side. As a sequela, right-sided partial hearing loss was observed by the patient, which has persisted in the subsequent follow-up examinations as well.

#### 3.2.3. Case 3

A 34-year-old male patient was admitted to the ED of the Albert Szent-Györgyi Clinical Center in Szeged, due to high fever (39.9 °C) and unconsciousness. In his past medical history, obesity (BMI > 30) and splenectomy was recorded 10 years ago due to a motorway accident. The patient was employed as a butcher in a local meatpacking plant, where he was reportedly injured on his hand by a sharp fragment of a pork bone, which occurred one day before admission. On arrival, the obese (BMI > 30) patient presented with no neck stiffness or meningism, nor peripheral stigmata of infective endocarditis. The patient had a high fever, chills, abdominal pain and watery diarrhea at his home; family members found the unconscious patient on the bathroom floor. XBLS (Extended Basic Life Support) and later ALS (Advanced Life Support) were started because of circulatory and respiratory failure. The patient was intubated and epinephrine was administered, which was followed by the return of spontaneous circulation. Cranial and abdominal CT scans and pulmonary angiography were carried out to rule out various pathologies. Cranial CT showed right-sided, multiple, acute ischemic lesions. On admission to the ICU, the patient presented with severe hypotension, sinus tachycardia and high fever, even after the administration of intravenous dopamine. Despite positive end-expiratory pressure (PEEP) appropriate oxygenation could not be achieved and respiratory and metabolic acidosis, severe hypoglycemia and hypokalemia were noted. Supportive therapy (K^+^-substitution, ventilation support and glucose) and subsequent high-dose vasopressor therapy only led to minor improvements. Duplicate blood cultures, urine samples, feces and sera for serology were taken and sent with urgency to the microbiology laboratory. Leukocyte cell count and C-reactive protein were 17.0 *×* 10^9^/L and 0.65 mg/dL, respectively. Laboratory results confirmed disseminated intravascular coagulation (DIC; suspected by bleeding from puncture sites and emergence of petechiae) and acute kidney and liver failure. The patient died from complications (DIC, multi-organ failure and acute respiratory distress syndrome) within 12 h of hospital admission.

Post-mortem examination verified DIC and multiple organ damage, microthrombosis and necrosis, predominantly in the lungs, liver, kidneys. The results of serology and microbiological analyses were received after the patient had already passed away. *S. suis* was detected in the blood culture samples previously submitted (time to positivity: 1 and 4 h, respectively), while viral serology only revealed a past EBV infection. *S. suis* was identified by MALDI-TOF MS (Bruker Daltonics, Billerica, MA, USA), with a reliable log score (>2.300). Identification was also complemented by 16S rRNA gene sequencing. Sequencing of the 16S rRNA gene confirmed that the strain belonged to the species *S. suis* with 99% nucleotide identity compared with GenBank accession no. NR036918, AF009487 and AM946016. The isolate was found to be the SS2 serotype. The strain was susceptible to β-lactam antibiotics and resistant to clindamycin, erythromycin and tetracycline. Resistance-determinants were verified using a PCR assay, indicating that our isolate with the tet(Q) and erm(B) genes.

## 4. Discussion

*S. suis* is a Gram-positive, facultative anaerobic bacterium, which is commonly found in the microbiota of pigs and they may also be found in other animals to a lesser extent [65]. The piglets may be healthy carriers or they may be afflicted by the presence of these pathogens. *S. suis* is normally found in the upper respiratory tract of pigs, mostly in the tonsils (tonsil carriage rate in piglets aged 4–6 months is around 32–50%), nasopharynx, gastro-intestinal, genital and alimentary tracts [66,67]. Transmission among pigs is generally the respiratory system in the form of direct nose-to-nose contact (horizontal transmission); however, vertical transmission through the genital tract during farrowing is also possible [68]. Susceptible animals may suffer from similar disease manifestations (meningitis, septicemia, pneumonia, endocarditis, or polyarthritis) as their human counterparts and the pathogenesis of the disease is also thought to be very similar [69]. Therefore, *S. suis* infections in animals are an important economic burden to the livestock-breeding industry. There are continent-wide programs aimed at the prevention of *S. suis* disease in livestock [70].

Infections caused by *S. suis*—which are mainly related to pigs—are causing zoonotic diseases of increasing frequency worldwide. The first human case was reported in 1968, with sporadic cases occurring worldwide in subsequent years. Nevertheless, the true significance of this zoonotic pathogen has been demonstrated after multiple outbreaks in Asia (e.g., China, Vietnam) associated with *S. suis* were reported [71]. Exporting pork goods (containing multiple *S. suis* serotypes) may lead to the development of more virulent strains through recombination or the exchange of mobile genetic elements between the local and foreign isolates [72]. A breach in the integrity of the skin is postulated to be one of the main routes of infection in Western countries; however, the precise route of transmission is not always identified [73]. Some cases have been linked to accidental inhalation of infected aerosols from infected carcasses, though *S. suis* is also found in faces and fomites of infected animals [1,2,15,73]. The prevalence of *S. suis* infections in humans has shown a notable increase in the last decade, with highest prevalence values in countries with a high pig density (i.e., Southeast Asia) [74]. There is a wide clinical variation in the presentation of *S. suis* infections in humans, but meningitis and sepsis are the most common and serious clinical manifestations of *S. suis* infections; these pathologies are usually accompanied with hearing loss in the surviving patients (which was also demonstrated in our Case 2) [75,76,77]. According to animal experiments, the human pathomechanism of hearing loss is likely from the invasion of *S. suis* to the perilymph, via the cochlear aqueduct, resulting in suppurative labyrinthitis [78]. This concomitant morbidity warrants the need of close monitoring and early adequate care in meningitis patients; in addition, the invasive nature of *S. suis* serotype 2 may have an important role in the development of these sequelae. The polysaccharide capsule containing sialic acid feature makes the organism highly invasive in entering the bloodstream and penetrating the blood-brain barrier [79]. One specific feature suggested for the European *S. suis* isolates is their predilection to the meninges and their frequent involvement in the etiology of purulent meningitis [80]. In a report published by Mancini et al., the first fatal human case of *S. suis* streptococcal toxic shock syndrome (STSS) in Italy was described and they have also published the draft genome of this strain. The patient was a 55-year-old male with Hodgkin’s lymphoma and a previous splenectomy, who presented with severe sepsis, DIC and MOF. The isolated strain from the blood culture samples was characterized by microbiological and molecular methods: the strain was found to be serotype 2, sequence type 1, had a plethora of virulence genes (*mrp*, *epf*, *sly*, *arc*A, *ofs* type 1 and *cps*2), a macrolide resistance determinant (*erm*B) and a rare variant of tetracycline resistance, involving efflux pumps and ribosomal protection (*tet*(O/W/32/O) and *tet*40) [81].

*S. suis* infections are not uncommon but, in Western countries, infections in humans are mostly reported sporadically [15]. Unfortunately, only a few reports exist related to epidemiological data on human *S. suis* infections from European countries (corresponding to the global prevalence by ~8–10%) [1,4]. There may be a correlation between European disease epidemiology and the reality that the invasive and virulent SS2 is frequently found in pigs on the continent [82]. Interestingly, North and Central American countries (USA, Canada, Mexico, Guatemala, Costa Rica and so on) reported the lowest number of human cases, while reports on *S. suis* affecting pigs are most numerous in the United States [25]. Some suggest strains in North America belonging to SS2 to be less virulent than strains in Europe and Asia. [51]. The first publication in Europe was from Denmark [11]; nevertheless, cases have been reported from various European countries including the United Kingdom [83], Germany [84], Belgium [85], France [86], the Netherlands [87], Switzerland [88], Sweden [89], Spain [90], Portugal [91], Italy [92], Greece [93], Austria [94], Hungary [95], Croatia [96] and Serbia [97] (it must be noted that several of these reports are only available in their native languages). Most European cases of *S. suis* infections in humans were reported from the Netherlands (*n* = 52), Poland (*n* = 22), France (*n* = 19), Germany (*n* = 19), the United Kingdom (*n*= 19) and Denmark (*n* = 12), while zoonoses caused by this pathogen occurred only sporadically in the remaining countries [1,2,25,51]. Surprisingly, no cases have yet been published in Russia, the Czech Republic, Slovakia or Romania, despite the fact that the economies of the previously mentioned four countries heavily rely on animal husbandry and the production of pork meat [6]. Overall, the most prevalent clinical syndrome described in human *S. suis* infections is meningitis (68.0%), followed by sepsis and toxic shock syndrome (25.0%), arthritis (12.9%), endocarditis (12.4%) and endophthalmitis (4.6%) [1,4,38]. The most common corresponding clinical symptoms of infection are generally similar to those of other bacterial pyogenic meningitis, including include headache, fever, vomiting, subjective hearing impairment, positive meningeal sings, the presence of skin rash, bacteremia, shock, acute renal failure, respiratory distress, MOF, changes in the common laboratory parameters (leukocytes, platelets, C-reactive protein) and findings in the CSF [1,4,38,51,52,53]. The onset of the disease is usually 1–4 days from coming in contact with the pathogen, and depending on the disease manifestation, the duration of illness may range between 1 and 24 days. If the patient is admitted, the length of their hospital stay is usually around two weeks (range: 1–46 days) [1,2,4,38]. The therapeutic approach of invasive *S. suis* infections is similar to those for other causes of bacterial meningitis/sepsis. Empiric antibiotic therapy (ceftriaxone ± vancomycin) should be initiated without delay, which must be re-evaluated after susceptibility-testing results become available [1,4,15,38]. Several case reports highlighted advantageous clinical outcomes and lower chance of hearing impairment if adjunctive dexamethazone is administered to the patients. In severe cases, renal replacement therapy, assisted ventilation and circulatory support must also be provided. Among recovered patients, 39% (31.0–47.8%) experience hearing loss, which is sensineural and affects the high frequency range; in ~75% of these cases, the hearing loss is irreversible [1,4,38,51,52,53]. Twenty-three percent (15.6–32.0%) of patients are also affected by vestibular dysfunction, which also significantly affects the quality of life of the individual [38].The low number of published human cases in these countries is presumably due to underdiagnosis and unawareness of the disease because the organism is often misidentified by microbiologists, which results in delay or inadequate treatment [25]. Misidentification may be very common in case of these bacteria; *S. suis* is frequently misidentified as *S. viridans*, while inappropriate results as *S. bovis*, *S. pneumoniae*, *E. faecalis* and *S. acidominimus* may also commonly occur [1,2,25,51,98]. In the case report of Tarini et al., the causative agent of meningitis for a 50-year-old male patient was identified as *S. mitis* with a 99% probability (based on the VITEK 2 Compact ID/AST system), while sequencing results from cerebrospinal fluid confirmed *S. suis* as the true pathogen [99]. The increasing use of modern diagnostic tools (e.g., PCR, MALDI-TOF MS) and methods available for use directly from clinical specimens may further aid the identification biochemically inactive or unreliably-identified isolates [100,101]. A very recently published report by Olearo et al. showed the first imported human case of *S. suis* infection in Switzerland: a 45-year-old woman bought and consumed raw pork meat imported from a small local farm in Moldova 24 h before flying back to Geneva, Switzerland. She was admitted to the local hospital in septic shock three days after ingestion of the pork meat and *S. suis* strains were isolated and next identified by 16 rDNA sequencing from blood cultures. Later on, the serotyping of the *S. suis* strain was identified as serotype 14, based on a high-resolution melting assay. According to EUCAST breakpoint criteria, the susceptibility tests revealed multi-susceptible isolates; subsequently, empiric antibiotic therapy (ceftriaxone) was de-escalated (to penicillin G for 22 days) until the patient has left the hospital [102]. Based on sequencing analysis, this human strain is not genetically close to strains originating from swine previously isolated in the country. The strain was sequence type ST1 (PubMLST ID: 2250), which was new type from Switzerland. The average nucleotide identity of this strain was between 94.0% and 99.9%, when compared to previously deposited full sequences of *S. suis* genomes [102].

As occupational contact with pigs or pork meat is one of the principal factors to consider (pig-related occupation was noted in ~38%, while other contact with pigs was reported in ~34% of published cases [4]), upholding the appropriate conditions of slaughterhouses is of utmost importance. Slaughterhouses are a good examples of epidemiological observatories, encompassing many aspects of the “One Health” perspective: they may be a source of infection in animals (resulting in local outbreaks and loss of income) and they have a role in facilitating zoonotic transmission from pigs to humans; thus, enforcing the compliance with national/international animal husbandry standards, environmental hygiene, cleaning and sanitation practices is crucial from a public health point of view. Continuous surveillance on *S. suis* carriage, serotype-distribution and the prevalence of associated diseases is also warranted among animals. From the human perspective, compliance with workplace regulations, the medical assessment of employees and education on workplace-related health risks is a responsibility of occupational medicine [1,2,4,15,25,35,41,53]. While swine-related occupancy is a well-known risk factor for human infections, interestingly, exposure to pigs or pork meat is not present in many of the published cases from European countries [1,2,15,25,41,53,81,82,83,84,85,86,87,88,89,90,91,92,93,94,95,96,97]. Although substantial new data on the incidence, clinical and microbiological characteristics, and risk factors for *S. suis* infection has been accumulated during recent years, there is not enough evidence to even estimate the prevalence of this infection on the continent. It was proposed that the principal transmission routes may significantly vary in Asian countries (oral transmission, through ingestion of raw meat) compared to the European region (transmission through injuries during meat processing) [1,2,15,25,41,53,81,82,83,84,85,86,87,88,89,90,91,92,93,94,95,96,97]. In fact, raw pork consumption was associated with more than four times (the reported odds ratio in the publication was 4.63) the risk of developing a *S. suis* infection (confidence interval: 2.94–7.29) [41]. However, in the report published by Manzin et al., a 68-year-old Italian patient developed a severe case of meningitis without having any contact with swine, other animals or any animal products; it was later discovered that the patient had an advanced-stage malignancy [103]. In most European countries, the infection rates among the exposed groups are poorly known as the diseases caused by *S. suis* are not notifiable, and only the United Kingdom and France consider *S. suis* infections in humans as an industrial-risk disease (affecting farmers, veterinarians, butchers, food processing workers and so on) [1,2,15,25,53,81,82,83,84,85,86,87,88,89,90,91,92,93,94,95,96,97]. While the colonization rate of *S. suis* in swine and wild boars has been extensively described (85–100%), there are very few reports on the human colonization rate with *S. suis*; most studies report on the risk-group population of people handling pork meat, with the colonization rates ranging between 0–10% [1,2,15,16,17,23,24,25,26,27,28,29,30,31,32,33,34,35].

The aim of our present study was to assess the spatio-temporal trends of invasive *S. suis* infections in Hungary between 2002 and 2019, based on the data available from the National Bacteriological Surveillance System. This is, to date, the first comprehensive study of human *S. suis* infections from Hungary, as even case reports were available only from microbiology and infectious disease congresses throughout Hungary. Overall, 34 patients were affected by invasive *S. suis* infections throughout the 18-year-long study period. In addition, we have presented three distinct cases, which showed the plethora of possible outcomes of *S. suis* infections: a patient recovered without any long-term consequences, one patient reported hearing loss as a long-term sequelae, while a third, splenectomised young patient has died suddenly and quickly from infectious complications. The relatively low frequency (2–3 cases/year) of invasive *S. suis* infections were highlighted in a period close to 20 years, despite the special characteristics of the country (being extensive consumers of alcohol and hard drinks due to several national traditions, an extensive agricultural sector which employs a relatively high proportion of the population, high levels of pork meat export and consumption). In addition, the prevalence of many underlying conditions responsible for higher risks of infections and immunosuppression (diabetes mellitus, alcoholism, smoking, obesity, cancers) are also pronounced in this country. Nevertheless, the average rate of infections was much higher than the reported prevalence in most Western European countries (0.35 vs. 0.01–0.10/100,000 persons). The geographical distribution did not show any correlation with the population of the respective towns, where the strains were isolated; however, most of the cases originated from provincial areas. Although older male patients were more likely to be affected, overwhelming generalizations on the risk population cannot be made, as there were persons from very different backgrounds, vocations and age groups among affected patients. Global efforts to develop effective vaccinations against invasive *S. suis* infections need to be strengthened, both for human subjects in the risk of environmental exposure (especially splenectomized patients), both for animals to reduce the economic burden of these infections in animal husbandry [104,105]. Bojarska et al. published one of the only available long-term reports on the prevalence of *S. suis* in East-Central Europe: as a part of the activities of the National Reference Centre for Bacterial Meningitis (NRCBM) in Poland between 2000–2013, *n* = 21 patients were affected by invasive *S. suis* infections [106]. Interestingly, during re-identification in the reference center, it was found that 48% of isolates were initially misidentified by the submitting microbiological laboratories. Most of the isolates were serotype 2, around half of the isolates were biofilm-producers and genes encoding for virulence factors (DNase, suilysin, extracellular protein factor, fibronectin-binding protein, muramidase-released protein, surface antigen one, enolase, serum opacity factor and pili) important in invasive disease were ubiquitous in these isolates. Their study concluded that the prevalence of *S. suis* diseases is most likely to be underestimated, as the country possesses a well-developed pork industry with a high output (over 10 million units) [106].

## 5. Conclusions

The results of our study highlight that clinicians and microbiologist need to be vigilant even in nonendemic areas, especially if the patients have a history of occupational hazards or having close contact with infected pigs even if they are immunocompetent. Educational efforts are needed among patients at increased infection risk, such as splenectomized patients or patients receiving immunosuppressive medication; these patients should avoid direct pig or pork contact when skin lesions—particularly on the hands—are present. During clinical situations, it is important to administer adequate therapy for patients with histories and symptoms suggestive of *S. suis* infections, even if laboratory verification of the diagnosis is not yet available or in case of negative cultures (due to previous administration of antimicrobials or misidentification). Infection prevention and control measures are currently the “best-buy” method available to control disease transmission, at least until an effective vaccine becomes available. From a public health perspective, educational campaigns on food safety could be an effective way to increase the understanding of the public regarding this illness, especially in regions, where there is strong association between traditional domestic pig slaughter and consumption of raw meat and meat products.

## Data Availability

All data generated during the study are presented in this paper.
